# Maladaptive Habitat Selection of a Migratory Passerine Bird in a Human-Modified Landscape

**DOI:** 10.1371/journal.pone.0025703

**Published:** 2011-09-30

**Authors:** Franck A. Hollander, Hans Van Dyck, Gilles San Martin, Nicolas Titeux

**Affiliations:** 1 Behavioural Ecology and Conservation Group, Biodiversity Research Centre, Earth and Life Institute, Université catholique de Louvain, Louvain-la-Neuve, Belgium; 2 Department of Environment and Agro-Biotechnologies, The Gabriel Lippmann Public Research Centre, Belvaux, Luxembourg; University of Western Ontario, Canada

## Abstract

In human-altered environments, organisms may preferentially settle in poor-quality habitats where fitness returns are lower relative to available higher-quality habitats. Such ecological trapping is due to a mismatch between the cues used during habitat selection and the habitat quality. Maladaptive settlement decisions may occur when organisms are time-constrained and have to rapidly evaluate habitat quality based on incomplete knowledge of the resources and conditions that will be available later in the season. During a three-year study, we examined settlement decision-making in the long-distance migratory, open-habitat bird, the Red-backed shrike (*Lanius collurio*), as a response to recent land-use changes. In Northwest Europe, the shrikes typically breed in open areas under a management regime of extensive farming. In recent decades, Spruce forests have been increasingly managed with large-size cutblocks in even-aged plantations, thereby producing early-successional vegetation areas that are also colonised by the species. Farmland and open areas in forests create mosaics of two different types of habitats that are now occupied by the shrikes. We examined redundant measures of habitat preference (order of settlement after migration and distribution of dominant individuals) and several reproductive performance parameters in both habitat types to investigate whether habitat preference is in line with habitat quality. Territorial males exhibited a clear preference for the recently created open areas in forests with higher-quality males settling in this habitat type earlier. Reproductive performance was, however, higher in farmland, with higher nest success, offspring quantity, and quality compared to open areas in forests. The results showed strong among-year consistency and we can therefore exclude a transient situation. This study demonstrates a case of maladaptive habitat selection in a farmland bird expanding its breeding range to human-created open habitats in plantations. We discuss the reasons that could explain this decision-making and the possible consequences for the population dynamics and persistence.

## Introduction

Habitat selection theory generally assumes that individuals are able to make optimal settlement decisions, thereby selecting the highest-quality habitats that are available in a heterogeneous landscape to maximize their fitness returns [1]. Such adaptive habitat choices are expected to produce an ideal free distribution [2] or similar patterns [3]. In this vein, the source-sink models of animal populations [4] are based on the assumption that individuals accurately evaluate habitat quality. Many studies have found strong empirical support so far [5]–[7], however, individuals are not always able to directly judge habitat quality in terms of fitness returns and, instead have to rely on environmental cues to guide their settlement decisions [8]. These environmental characteristics need to be reliable at the time of habitat choice, but should also reflect habitat quality at some later time [9]–[10]. For instance, migratory birds are often time-constrained in the selection of their breeding sites and have to use indirect cues to determine local habitat quality. The use of environmental cues allows the migratory animal a fast assessment of habitat quality. It has been shown that these organisms may rely upon a host of proximate cues reflecting the environmental conditions that will ultimately affect fitness, such as the vegetation structure and phenology [11], food availability [11]–[13], anti-predation shelters [14] or social attraction [15].

Human-driven environmental changes may induce a mismatch between the attractiveness of the habitats (i.e. the response of the individuals to the cues) and their quality (i.e. the fitness returns for the individuals) [16]–[20]. As a consequence, anthropogenic activities may bring some organisms to prefer lower-quality habitats although higher-quality options are available. Such an ecological trapping may occur when (1) cues for habitat selection become uncoupled from habitat quality (i.e. unchanged habitat attractiveness in combination with decreasing habitat quality), (2) changing (or newly created) habitat selection cues increase the attractiveness of some habitats with no effect on habitat quality, or (3) both conditions are combined [19], [21]. Although ecological trapping is a behavioural, individual process [19], this phenomenon may have negative population-level consequences and reduce the likelihood of population persistence [16], [22].

Ecological trapping is an extreme situation of non-ideal habitat selection, with a negative relationship between habitat preference and quality [16]–[21], therefore field studies need to determine: (1) a quantitative evaluation of habitat preference at the individual level and (2) the ultimate influence of habitat quality relative to several fitness attributes [19], [21]. Habitat preferences should ideally be estimated with choice experiments, but this often proves to be unachievable in the field for logistical reasons. Hence, Robertson & Hutto [19] listed some surrogate measures of preference: order of settlement (in migratory organisms), distribution of dominant individuals, site fidelity and temporal variation in population size. To date, few studies examining the existence of an ecological trap have investigated the link between habitat characteristics, fitness and individual preferences using multiple measures of habitat preference over a substantial time period.

Here, we examined the link between habitat preference and fitness in the Red-backed shrike (*Lanius collurio*), a migratory passerine that occupies two distinct types of breeding habitats in northwest Europe. The Red-backed shrike is typically considered as a farmland bird inhabiting open areas under a management regime of extensive farming [23], [24]. However, during the last few decades, the species has also colonised novel and artificially created open areas in Norway Spruce (*Picea abies*) plantations that result from recent changes in forest harvesting techniques [25]–[28]. In most cases, Spruce forests are managed with large-size cutblocks in even-aged plantations [29]. It has been suggested that in an evolutionary past, the shrikes (and other birds of this ‘farmland’ community) lived in open areas created by natural disturbances in forests (e.g. windfalls or wildfires [23], [30]) and that they secondarily shifted to extensively managed, heterogeneous farmland since the Neolithic period [31]. So, the early-successional regeneration areas following harvesting activities in Spruce plantations constitute a novel habitat compared to farmland, and this habitat is likely to share some features with the historic natural woodland breeding habitat [32]. Hence, open areas in Spruce plantations may function as a potentially attractive environment to the species. We used multiple measures of individual habitat preference (i.e. settlement pattern and distribution of dominant individuals) and fitness components (i.e. reproductive performance) in both habitat types over several years to test whether habitat selection in the Red-backed shrike is based on adaptive decisions, or alternatively, whether there is support for ecological trapping in a human-altered landscape undergoing quick changes.

## Materials and Methods

### Study species

The Red-backed shrike (hereafter “shrike”) is an insectivorous long-distance migratory bird that has a wide breeding range across the Western Palaearctic and overwinters in southern Africa [23]. Males arrive on the breeding sites from late April to late May, and on average two days before females [25], [33], [34]. The breeding season is typically short (May-July) producing a single clutch, although there can be a replacement clutch in case of breeding failure [33], [35].

### Study areas

Based on prior knowledge of the distribution of shrikes in South Belgium [27], two study areas of 400-km^2^ each were selected (centres of study areas 1 and 2 are 50°14′N 5°50′E and 49°49′N 5°39′E, respectively), representing a mosaic of farmland and woodland breeding habitats (i.e. farmland with bushes, hereafter “farmland” and early-successional regeneration areas in Spruce plantations, hereafter “woodland”). The farmland areas are mainly covered by pastures and hay meadows and, to some extent, by fields. In South Belgium, Spruce plantations were initiated in 1850 and over the last century their cover has increased fourfold [36]. The current coverage of Spruce plantations is estimated to be 30% of all forested areas [36].

### Habitat preference

Local population density may not necessarily reflect habitat preference [37], [38]. In migratory birds, one of the closest alternatives to choice experiments is the order of settlement [19], [21]. During the winter of 2007–2008 and in both study areas, all potential sites for the establishment of shrike territories in farmland (F sites) and woodland (W sites) were identified based on the presence of the main habitat requirements for the species (e.g. nest sites and foraging areas [24]). During three consecutive breeding seasons (2008, 2009 and 2010), the F and W sites in both study areas (N = 118) were visited on a bi-daily basis from late April to late May. To avoid bias in the occupancy histories, the same proportion of F and W sites was visited each day and the order in which sites were visited was randomized. During a 15-minute survey within each site, we checked the presence of territorial males using both visual and auditory cues. Males are easy to survey with high detection probability due to their conspicuously territorial signalling behaviour in springtime. Once settled, males attract females by repetitive advertising calls on perches and by typical fluttering flights [23]. Males are highly territorial: they defend their territorial resources against intruders and this may induce escalated conflicts [39]. As assumed in other (long-term) studies on arrival dates in the Red-backed shrike [34], we considered the date of first detection in a site as a reliable estimate of the arrival date.

The distribution of dominant individuals among habitat types is another alternative measure of habitat preference since the most dominant individuals are expected to be found more often in the preferred habitat type [19]. In shrikes, adult wing length has been shown to be positively related to age and may relate to dominance [40]. Breeding individuals were captured all along the breeding season with bird traps using mealworms as lures. The outermost primary wing length was measured (precision: ±0.1 mm) and used as a first surrogate of adult dominance [40]. We also examined the size of black eye-stripes (facial mask) in males because in other species it has been shown that this morphological trait is an indicator of male quality and dominance [41]. The male mask size was measured from digital pictures (digital camera Nikon D70 with Nikkor 50 mm lens) taken at standardized focal distance (20 cm) using ImageJ analysis software.

### Reproductive performance

In all F and W sites where adult presence was recorded in 2008, 2009 and 2010, we searched for nests from mid-May to late July on a regular basis (i.e. every 2–5 days depending on the progress of the reproduction). During these surveys, the male and/or female feeding behaviour provided us with reliable indication of the nest location. Because adults are likely to abandon their brood if disturbed during incubation [25], [33], nests were mostly visited during the nestling period (i.e. when feeding visits of both male and female were observed). Nestling age was estimated with a 1-day precision by means of visual comparisons with feather characteristics of age-known broods [25]. The number of nestlings and their body condition were mostly measured at the age of 12 days (range: 11–15 days). Measurements included tarsus length and outermost primary wing length (digital callipers; precision: ±0.01 mm), as well as body mass (laboratory balance; precision: ±0.1 g). Since these measurements were strongly correlated, the first component of a principal component analysis (PCA) was used as a synthetic measure of nestling body condition (variance explained: 89%).

A double brood is considered as an exceptional event in the Red-backed shrike [23] and this never occurred in our study. Predation by corvid robbing may, however, be considerable in this passerine species during the incubation or the nestling period, and is the main reason for breeding failure [35]. During our surveys in the sites every 2–5 days over the course of the breeding season, we recorded the interruption of feeding visits by both the male and female and we attributed this to a breeding failure related to predation or accidental loss due to harsh weather conditions. After a breeding failure, the same parents renested within the same territory site, thereby excluding within-season movements between territories [42].

We used the following measures of reproductive performance in first or replacement clutches (hereafter “clutch sequence”: 1 or 2 respectively) for each breeding pair in each year: nest success (i.e. production of at least one fledgling), brood size (i.e. number of nestlings older than 12 days) and nestling body condition (i.e. combination of tarsus length, wing length and body mass). The use of a series of fitness-related parameters that incorporate several components of season-long reproductive performance (i.e. offspring quantity and quality in first and replacement clutches) is well suited to evaluate habitat quality at the individual level [43].

### Weather conditions

Weather conditions may impact nestling body condition because of weather-related insect prey activity [44]. A possible general trend in weather conditions over the course of the breeding season may therefore confound the relationship between habitat preference and fitness consequences, as some estimates of preference in our study (arrival date) are related to breeding time. To control for this in our analyses, the following climatic variables were used to reflect regional weather conditions during a 5-day period before the nestling measurements: wind velocity (Bf), precipitation (mm), ambient temperature (°C) and solar radiation (Watt/m^2^). They were derived from 15-minutes resolution datasets (data sampled between 7h30 am and 7h00 pm) that are freely available from nearby weather stations in Luxembourg [45]. The stations Schimpach and Roodt are at 10 km and 21 km from the study areas 1 and 2, respectively.

### Statistical analysis framework

We first examined how the structural characteristics of territories in different habitats (i.e. F versus W sites) were linked to individual-level measures of habitat preference (arrival date and dominance of males) and, second, whether there was a link between the habitat and the measures of season-long reproductive performance (nest success, brood size and nestling body condition).

We used generalized linear mixed models (GLMMs, Maximum Likelihood estimations) to relate arrival date of males (N = 300), male wing length (N = 102), male mask size (N = 94), nest success (N = 438), brood size (N = 243) and nestling body condition (N = 1001) to the type of habitat (class variable: F/W sites). Sample sizes differed between different models due to missing values and because of the contrasting level of the response variable (male, nest or nestling). We used normal distribution models with identity link, except for the models of nest success where we used binomial distribution models with logit link. Apart from the models of nestling body condition, the territory site identity was always defined as a random factor because (1) the same site can be occupied in several years and (2) more than one breeding pair or individual male can occupy the same site. In the models of nestling body condition, the nest identity was defined as a random factor because the measures on the nestlings from the same nest are not independent. In all models we included year, study area and their interaction with F/W sites as independent variables to investigate the consistency of the results over time and in space. Because the settlement of a male may be triggered by social information relative to the presence of other males in the neighbourhood [15], we calculated, for each male in each year separately, the total number of males settled within a radius of 500 m and we included this territory aggregation factor (hereafter “aggregation”) as an independent variable in the models of arrival dates. To reduce the influence of prior occupancy on settlement decisions, colour-ringed males showing site fidelity from year t-1 to year t (N = 8) were excluded from the analysis of arrival dates. The clutch sequence and its interaction with F/W sites were included in the models of nest success, brood size and nestling body condition to account for possible differences in reproductive performance between first and replacement clutches. In addition, we used nestling age and weather conditions as covariates in the models of nestling body condition to control for their possible effect on the nestling measurements. Complete brood failures due to predation or harsh weather conditions after the replacement clutch (N = 20) were excluded from the models of brood size and nestling body condition.

Model selection procedures were implemented to evaluate the strength of evidence for the relative influence of the different independent variables included in the models [46], [47]. Full models included the whole set of independent variables and covariates separately for arrival date of males, male wing length, male mask size, nest success, brood size and nestling body condition. All possible combinations of variables (hereafter “candidate models”) were then derived from the full models. Interactions were only incorporated in a candidate model when both main effects were also included. The covariates were forced in all candidate models.

Information-theoretic multimodel inference was used based on the Akaike's Information Criterion corrected for small sample sizes (AIC_c_). The differences in AIC_c_ between the i candidate models (Δi) were used to rank them from best to worst. A Δi value <2 (relative to the best model associated with the smallest AIC_c_) was used as a threshold for a model to be considered as having support. The relative support for the alternative models was obtained by scaling them according to their AIC_c_ weight [46]. The relative importance of a variable (hereafter *w_+_*) was estimated by summing the AIC_c_ weights across all candidate models in which the variable occurred. Since the prevalence of the variables in the set of candidate models (ν) varied from one variable to the other and because this prevalence restricts the *w_+_* values associated with the variables [46], the ν values are reported as a baseline reference along with the *w_+_* values. The model-averaged parameter estimates (β), the estimate precision (unconditional standard errors, hereafter S.E.) and the *w_+_* values inform on the strength of importance of each variable [46]. In order to facilitate the interpretation, these estimates were converted into percentages relative to the average value of the response variables (see Δ[difference between two levels of a variable] hereafter). All analyses were performed with R 2.8 and SAS 9.1 (PROC MIXED) software.

## Results

Table 1 summarizes the different sets of supported models (Δi<2) related to habitat preference and reproductive performance derived from the model selection procedures.

**Table 1:** Set of supported (Δi<2) and best non-supported (Δi>2, between brackets) models for habitat preference (arrival date, wing length and mask size of males) and reproductive performance (nest success, brood size and nestling body condition) measures along with their respective support (AIC_c_ weight).

Response	Supported and (best non-supported) models	K	Log Likelihood	Δi	AIC_c_ weight
**Arrival date of males**	F/W+Year	6	−973.03	0.00	0.26
	F/W+Year+Study area	7	−971.99	0.03	0.25
	F/W+Year+Aggregation	7	−972.69	1.43	0.13
	F/W+Year+Study area+Aggregation	8	−971.80	1.76	0.11
	(F/W+Year+Study area+F/W*Study area)	(8)	(−971.99)	(2.14)	(0.09)
**Male wing length**	F/W	4	−220.57	0.00	0.41
	F/W+Year	6	−219.13	1.59	0.19
	F/W+Study area	5	−220.31	1.69	0.18
	(F/W+Year+Study area)	(7)	(−218.93)	(3.51)	(0.07)
**Male mask size**	F/W+Year+F/W*Year	9	−946.23	0.00	0.25
	F/W+Year	7	−948.63	0.06	0.24
	F/W+Year+Study area	8	−947.62	0.38	0.20
	F/W+Year+Study area+F/W*Year	10	−945.66	1.30	0.13
	(F/W+Year+Study area+F/W*Study area)	(9)	(−947.61)	(2.77)	(0.06)
**Nest success**	F/W+Year+Study area+Clutch sequence+F/W*Study area	8	−246.81	0.00	0.37
	F/W+Year+Study area+Clutch sequence	7	−248.57	1.45	0.18
	F/W+Year+Study area+Clutch sequence+F/W*Study area+F/W*Clutch sequence	9	−246.66	1.79	0.15
	(F/W+Year+Study area+Clutch sequence+F/W*Clutch sequence)	(8)	(−248.57)	(2.92)	(0.09)
**Brood size**	F/W+Clutch sequence+Year+F/W*Clutch sequence	8	−343.42	0.00	0.34
	F/W+Clutch sequence+Year	7	−345.09	1.23	0.19
	F/W+Clutch sequence+Year+Study area+F/W*Clutch sequence	9	−343.30	1.90	0.13
	(F/W+Clutch sequence+Year+Study area)	(8)	(−345.05)	(3.26)	(0.07)
**Nestling body condition**	F/W+Clutch sequence	10	−1289.84	0.00	0.48
	(F/W)	(9)	(−1292.08)	(2.43)	(0.14)

Δi refers to the differences in AIC_c_ between the model and the best candidate model associated with the smallest AIC_c_. The number of parameters (K) is reported for each model.

Response variables: Arrival date of males = arrival dates of males in springtime, Male wing length = outermost primary wing length in males, Male mask size = size of black eye-stripes in males, Nest success = production of at least one fledgling, Brood size = number of nestlings older than 12 days, Nestling body condition = PCA-based combination of nestling tarsus length, wing length and body mass.

Fixed effects: F/W = farmland (F) versus woodland (W) sites, Clutch sequence = first versus replacement clutches, Year = 2008, 2009 or 2010, Study area = 1 or 2, Aggregation = number of males settled within a radius of 500 metres.

Random effects: territory site identity (for arrival date, male wing length, nest success and brood size) or nest identity (for nestling body conditions).

### Habitat preference

#### Arrival date of males

**Figure 1:**
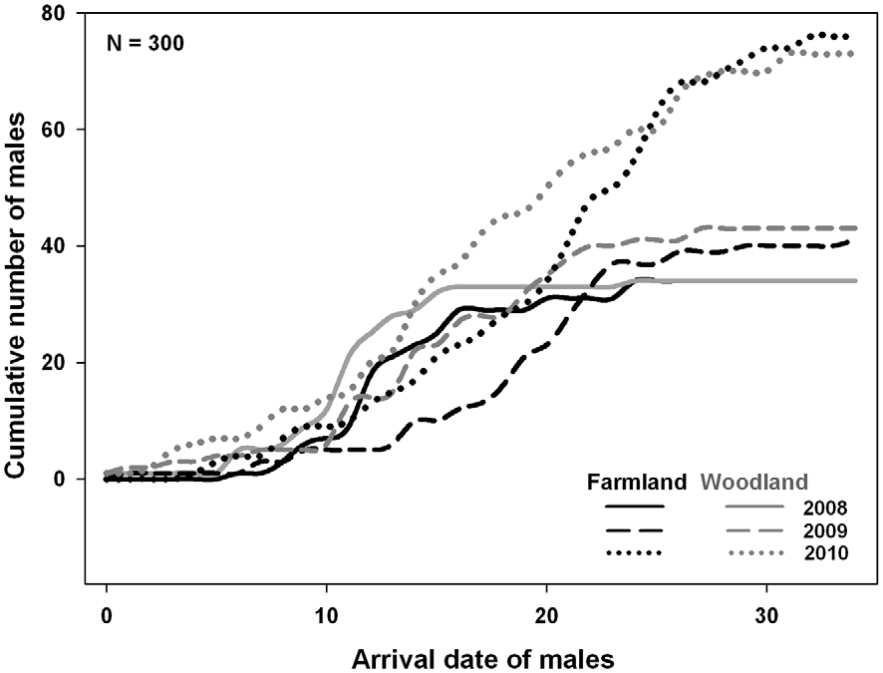
Cumulative number of territories occupied by males along the breeding season in farmland and woodland sites. The date corresponding to the first arrival within each year is set to 0.

There was strong support for the effect of year on male arrival date (Table 2, *w_+_* = 100%, Δ[2008–2009] = −20%, Δ[2008–2010] = −36%) and for overall differences in arrival date between F and W sites (*w_+_* = 100%, Δ[F–W] = 17%). On average, males arrived more than 3 days earlier in W sites compared to F sites. A low AIC_c_-based weight was found for the territory aggregation variable (*w_+_* = 31%): the settlement of males was earlier when other males were settled in the neighbourhood, but this influence was only weakly supported. Arrival dates of males were rather similar in both study areas (*w_+_* = 56%, Δ[1]–[2] = −3%). The weak support for the interactions (all *w_+_*<14%) indicates that the earlier arrival of males in W sites was consistent over time and space. Fig. 1 shows the cumulative number of males in F and W sites over the course of the breeding season. In each year, early-arriving males occupied W sites more frequently than F sites at the beginning of the breeding season and this pattern was gradually inverted during the progress of the season. As a consequence, the cumulative curves of male arrival (Fig. 1) were found to follow different distributions in F and W sites (Kolmogorov-Smirnov Two-Sample Tests, 2008: p = 0.03, D = 0.34; 2009: p = 0.02, D = 0.35; 2010: p = 0.02, D = 0.27).

**Table 2:** Results of the AIC_c_-based multimodel inference procedure examining the variations in habitat preference relative to the independent variables.

		Arrival date of males				Male wing length				Male mask size			
Fixed effect	ν	*w*_+_	β	S.E.	Effect	*w*_+_	β	S.E.	Effect	*w*_+_	β	S.E.	Effect
*(Intercept)*	*100*	*100*	*13.89*	*1.12*		*100*	*94.63*	*0.42*		*100*	*43555*	*1532*	
**F/W** (W)	69	100	−3.58	1.00	Settlement of males earlier in woodland sites	100	1.47	0.48	Male wing length longer in woodland sites	92	1819	1907	Male mask size is larger in woodland sites
**Year** (2009)	62	100	4.40	1.06	Settlement of males earlier in 2008 and later in 2010	35	−0.14	0.22		100	4658	2165	Male mask size larger in 2010 and smaller in 2008
**Year** (2010)			6.31	1.00			−0.31	0.27			9429	1756	
**Study area** (2)	62	56	0.67	0.61		36	0.09	0.18		48	823	793	
F/W*Year (W, 2009)	23	13	−0.07	0.27		7	0.09	0.11		41	2455	1920	Larger male mask size in woodland is more pronounced in 2009
F/W*Year (W, 2010)			−0.16	0.28			0.05	0.08			441	1278	
F/W*Study area (W, 2)	23	14	0.02	0.24		9	0.04	0.09		10	−41	272	
**Aggregation**	50	31	−0.06	0.09		-	-	-		-	-	-	

The AIC_c_-weighted relative importance (*w_+_*), the model-averaged estimate (β) and their unconditional standard error (S.E.) are reported for each parameter (main effects and interactions), as well as their respective prevalence in the candidate models (ν). The ν and w_+_ values range between 0 and 100%. The parameter estimates refer to the level indicated between brackets as a baseline. The interpretation of each effect is provided in case of AIC_c_-based support.

Response variable: Arrival date of males (days) = arrival dates of males in springtime, Male wing length (mm) = outermost primary wing length in males, Male mask size (mm^2^) = size of black eye-stripes in males.

Fixed effects: F/W = farmland (F) versus woodland (W) sites, Year = 2008, 2009 or 2010 and Study area = 1 or 2, Aggregation = number of males settled within a radius of 500 metres.

Random effects: territory site identity.

#### Male wing length

**Figure 2:**
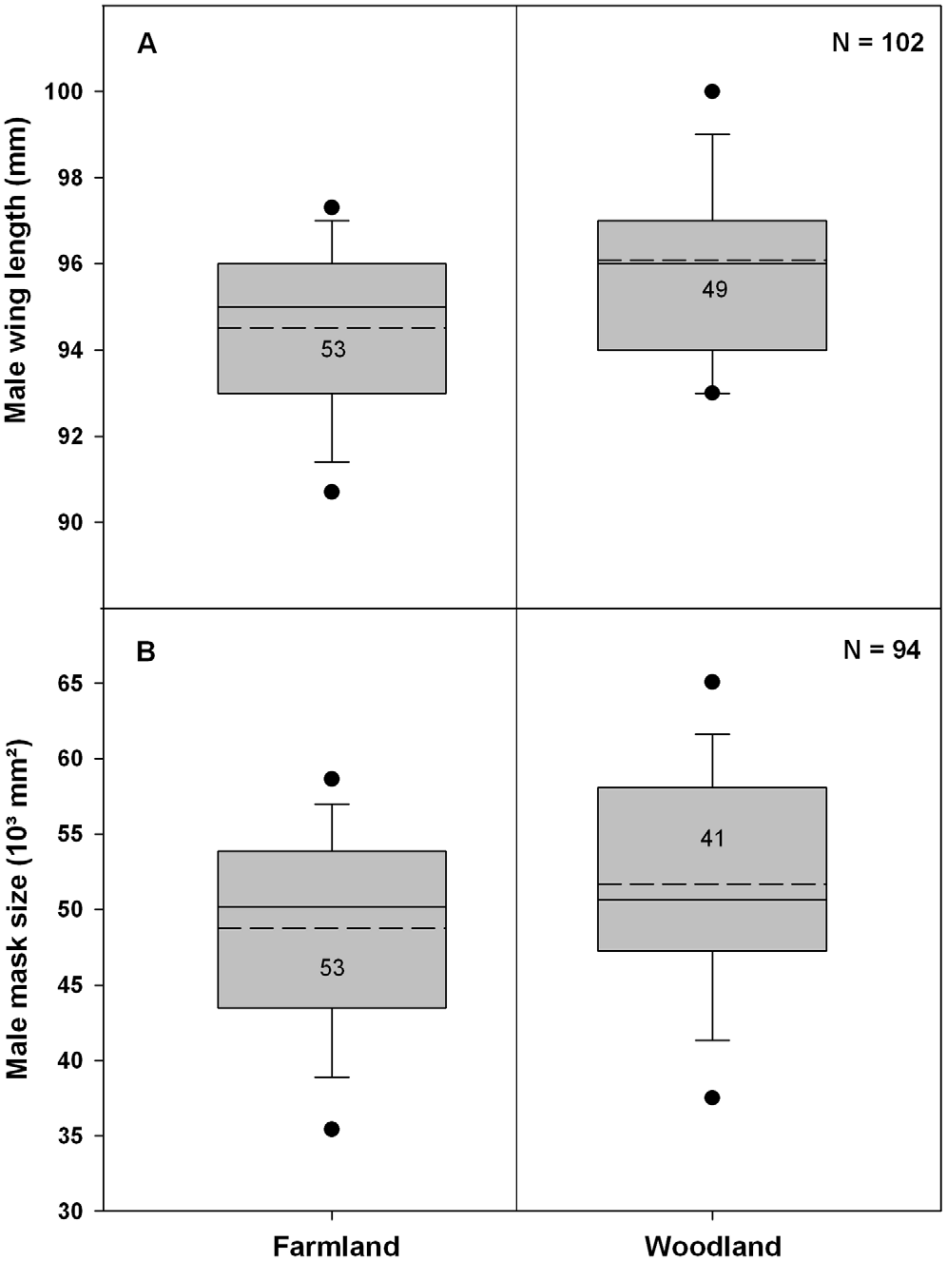
Dominance-related traits of males in farmland and woodland sites. Box-and-whisker plots and quartile distributions (5^th^/95^th^ percentile [•], mean [−] and median [--]) for A: male wing length and B: male mask size in farmland and woodland sites.

On average, male wings were longer in W sites than in F sites (Table 2, *w_+_* = 100%, Δ[F–W] = −16%, Fig. 2a), and the influence of year and study area was not supported (all *w_+_*<36%). Interestingly, there was also a slight trend for longer wing length in early arriving males (simple linear regression, p = 0.0002, F_1,87_ = 15.4, R^2^ = 0.14).

#### Male mask size

The size of black eye-stripes in males was larger in W than in F sites (Table 2, *w_+_* = 92%, Δ[F–W] = −5%, Fig. 2b), although there was more among-year variation in this dominance-related trait (*w_+_* = 100%, (Δ[2008–2009] = −12% and Δ[2008–2010] = −18%). The larger male mask size in W sites was more important in 2009 (F/W*Year: *w_+_* = 41%, Δ[F–W] = −12%), than in 2008 (Δ[F–W] = −2%) and 2010 (Δ[F–W] = −2%).

### Reproductive performance

#### Nest success

**Figure 3:**
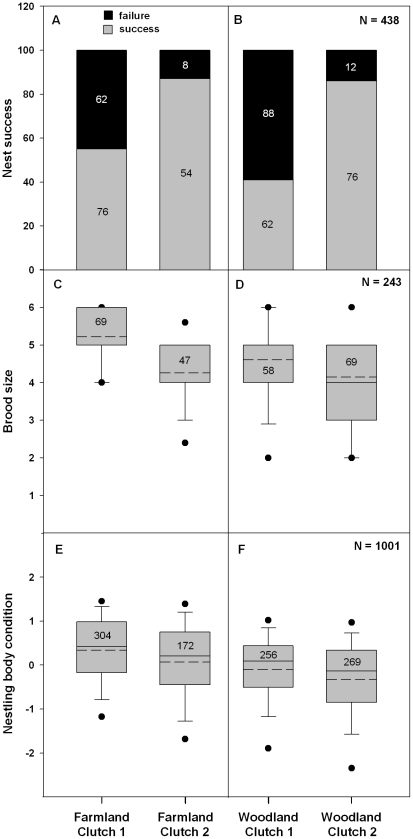
Season-long reproductive performance in farmland and woodland sites. Proportion of breeding attempts associated with success (grey) and failure (black) in farmland (A) and woodland (B) sites for the first (1) and replacement (2) clutches. Box-and-whisker plots and quartile distributions (5^th^/95^th^ percentile [•], mean [--] and median [−]) for brood size (C, D) and nestling body condition (E, F) in farmland and woodland sites for the first and replacement clutches. The nestling body condition data used in the plots are the residuals resulting from a linear regression against nestling age and weather conditions to remove the effect of the covariates forced in the analysis. The number of nests (for nest success and brood size) or nestlings (for nestling body condition) is indicated in the boxes.

The nest success of shrikes was markedly higher for a replacement clutch than for a first clutch (Table 3, *w_+_* = 100%, Δ[1]–[2] = −39%, Fig. 3a–b). On average, nest success was also higher in F than in W sites (*w_+_* = 97%, Δ[F–W] = 8%, Fig. 3a–b) and this difference is more pronounced in first clutches (Δ[F–W] = 14%) compared to replacement clutches (Δ[F–W] = 1%) (F/W*Clutch sequence, *w_+_* = 30%, Fig. 3a–b). There is also support for a difference in nest success among years (*w_+_* = 95%, Δ[2008–2009] = −12%, Δ[2008–2010] = −9%) and study areas (*w_+_* = 95%, Δ[1]–[2] = −8%). Importantly, the higher nest success in F sites was consistent over time (F/W*Year, *w_+_* = 10%). The difference between F and W sites was more markedly pronounced in study area 2 compared to study area 1 (F/W*Study area, *w_+_* = 61%).

**Table 3:** Results of the AIC_c_-based multimodel inference procedure examining the variations in season-long reproductive performance relative to the independent variables.

		Nest success				Brood size				Nestling body condition			
Fixed effect	ν	*w*_+_	β	S.E.	Effect	*w*_+_	β	S.E.	Effect	*w*_+_	β	S.E.	Effect
*(Intercept)*	*100*	*100*	*−0.85*	*0.38*		*100*	*4.80*	*0.22*		*100*	*0.25*	*0.68*	
**F/W** (W)	77	97	−0.32	0.37	Nest success higher in farmland sites	100	−0.55	0.24	Brood size higher in farmland sites	100	−0.41	0.09	Nestling body conditions higher in farmland sites
**Clutch sequence** (2)	63	100	2.11	0.34	Nest success higher in replacement clutches	100	−0.83	0.21	Brood size higher in first clutches	74	−0.19	0.09	Nestling body conditions higher in first clutches
**Year** (2009)	63	95	0.93	0.32	Nest success higher in 2009 and lower in 2008	97	0.36	0.21	Brood size higher in 2010 and lower in 2008	12	0.014	0.02	
**Year** (2010)			0.65	0.30			0.57	0.19			0.055	0.06	
**Study area** (2)	63	95	0.79	0.35	Nest success higher in study area 2	34	−0.01	0.05		20	0.027	0.03	
F/W *Clutch sequence (W, 2)	26	30	0.11	0.18		64	0.30	0.20		10	0.003	0.02	
F/W*Year (W, 2009)	26	10	0.01	0.07		17	−0.05	0.08		1	−0.003	0.004	
F/W*Year (W, 2010)			0.01	0.06			−0.002	0.06			−0.001	0.003	
F/W*Study area (W, 2)	26	61	−0.50	0.33	Higher nest success in farmland is more pronounced in study area 2	9	−0.009	0.03		3	0.001	0.005	

The AIC_c_-weighted relative importance (*w_+_*), the model-averaged estimate (β) and their unconditional standard error (S.E.) are reported for each parameter (main effects and interactions), as well as their respective prevalence in the candidate models (ν). The ν and w_+_ values range between 0 and 100%. The parameter estimates refer to the level indicated between brackets as a baseline. The interpretation of each effect is provided in case of AIC_c_-based support.

Response variables: Nest success = production of at least one fledgling, Brood size = number of nestlings older than 12 days, Nestling body condition = PCA-based combination of nestling tarsus length, wing length and body mass.

Fixed effects: F/W = farmland (F) versus woodland (W) sites, Clutch sequence = first versus replacement clutches, Year = 2008, 2009 or 2010, Study area = 1 or 2.

Random effects: territory site identity (for nest success and brood size) or nest identity (for nestling body condition).

#### Brood size

The AIC_c_-based model selection procedure provided strong evidence for an effect of clutch sequence on brood size (Table 1): an overall difference of almost one nestling per nest was found between first and replacement clutches (Δ[1]–[2] = 14%) (Table 3, *w_+_* = 100%, Fig. 3c–d). Clear evidence was also found for a difference in brood size between F and W sites, with on average half a nestling increase in nests located in F sites (*w_+_* = 100%, Δ[F–W] = 9%). Furthermore, there was an interaction between F/W and clutch sequence (*w_+_* = 64%) suggesting that the difference in brood size is particularly marked in first clutches (Δ[F–W] = 19%) and less pronounced in replacement clutches (Δ[F–W] = 5%). There was some support for a year effect on brood size (*w_+_* = 97%, Δ[2008–2009] = −7%, Δ[2008–2010] = −12%), but the number of nestlings was similar is both study areas (*w_+_* = 34%). A higher brood size in F sites was consistently found over time and space (F/W*Year and F/W*Study area, all *w_+_*<17%).

#### Nestling body condition

One single best model indicated strong support for a difference in nestling body condition between F and W sites and, to a lesser extent, for an effect of clutch sequence (Tables 1 and 3, Fig. 3e–f). Better nestling body condition was observed in F sites (Table 3, *w_+_* = 100%, Δ[F–W] = 6%) and for first clutches (*w_+_* = 74%, Δ[1]–[2] = 3%). The effects of F/W and clutch sequence on nestling body condition were additive as there was no support for an interaction between both variables (*w_+_* = 10%). In addition, the influence of year and study area on nestling body condition was not supported.

## Discussion

Over recent decades, changes in forest harvesting techniques have created rotation systems of large, open areas in plantation forests [29], [36]. Interestingly, some similar bird assemblages (and other wildlife populations [48], [49]) are found in farmland sites and in early-successional vegetation following the harvesting activities [26]. Using the Red-backed shrike as a model organism that occupies both habitat types in a mosaic of farmland and woodland sites, we demonstrate a preference for Spruce plantations over farmland sites, even though reproductive performance was higher in farmland.

On average, territorial males occupied the woodland sites before the farmland sites and dominant males (i.e. males with longer wing length and, to a lesser extent, larger mask size) were found more often in woodland sites. As order of settlement and distribution of dominant individuals are considered as individual-level measures of habitat preference [19], these results indicate a preference for the novel woodland environment rather than for the traditionally used farmland. Early arrival has been shown to be beneficial for territory acquisition and reproductive performance in territorial, migratory birds [50], but the costs for arriving early should be considerable [51] and the individuals with the best flight performance and condition arrive earliest at the breeding sites [52], [53]. In the Red-backed shrike, wing length relates to the age of the bird [40]. So, older males associated with better flight abilities (wing length) and higher quality (mask size) could reach the breeding sites before younger and less experienced males [54], resulting in the earlier, dominant males preferentially settling in woodland sites.

Unlike most studies (see [55]), reproductive performance was examined according to a hierarchical approach combining nest success, fecundity (brood size) and offspring quality (nestling body condition) in first and replacement clutches. First, the proportion of successful first clutches was considerably higher in farmland sites, whereas a higher amount of replacement clutches was recorded in woodland sites. This is of great importance as replacement clutches produced, on average, one nestling less compared to first clutches. Replacement clutches are known to be costly in additional energy use, thereby explaining the reduced brood size [56], [57]. Second, fecundity in first clutches was higher in farmland sites than in woodland site. Third, individual nestling body condition was better in farmland sites and this pattern was consistent for first and replacement clutches. As both the number of fledglings and their body condition were higher in farmland sites, a general life-history trade-off between quantity and quality of offspring [58], [59] can be ruled out in our study.

The observed mismatch between habitat preference and fitness-related parameters is in agreement with the Robertson & Hutto's definition of an ecological trap [19]: our results on the Red-backed shrike indicate the existence of maladaptive habitat selection in mixed farmland-woodland landscape under intense human-use where the species has recently colonised open areas in Spruce plantations. Importantly, this study shows that the behaviour of the shrike is maladaptive over several years and so demonstrates that the identified ecological trap does not simply represent transient and exceptional conditions. Ecological trapping has recently attracted much attention in anthropogenic environments, but few studies have provided empirical evidence so far [19]. We suggest here that the novel environment in a human-modified farmland-woodland landscape may induce the preference for lower-quality habitats in the Red-backed shrike and possibly in other birds. However, important points need further study. First, reproductive performance is only one component of fitness and estimation of survival rates is important for a complete capture of habitat quality [43]. Second, population growth rates need to be evaluated separately for farmland and woodland before we can argue that woodland sites constitute an attractive sink. Third, the behavioural processes operating during habitat selection and the cues used by individuals to select their breeding habitat remain unknown.

Use of reproductive performance to estimate habitat quality may receive criticism because there could be a possible trade-off between reproduction and survival [60]. Arlt and colleagues [61] have recently shown that the Northern Wheatear (*Oenanthe oenanthe*) occupies structurally different habitat types in central Sweden and, although habitats differ with respect to reproductive performance, differences in habitat-specific population growth are largely due to differences in adult and first-year survival rates. Although nestling body condition correlates strongly with first-year survival rates in passerine species [62], [63], this reproductive performance measure was not considered in the extensive work of Arlt and colleagues. With our hierarchical examination of reproductive performance nestling body conditions in shrikes were found to be higher in farmland and so, we can reasonably assume that poorer reproductive performance in woodland sites may not be compensated for by higher first-year survival rates. We acknowledge, however, that a more complete estimation of fitness based on long-term reproduction and survival data (also on adults, see [64]) is needed to determine local recruitment, lifetime reproductive performance and population growth rates. Such a complete estimate of individual fitness would better determine whether shrikes exhibit a preference for sink habitats associated with negative consequences for population dynamics or prefer the lower-quality of two source habitats. So far, population declines induced by individual, maladaptive habitat choices have been validated only on a theoretical basis [9], [16], [22], and empirical evidence is lacking. Arlt and colleagues showed that wheatears in Sweden display non-ideal habitat selection because individuals fail to discriminate between source and sink habitats, but their results were not in agreement with an ecological trapping situation where lower-quality habitats are preferred over higher-quality ones [61], [65]. An estimation of survival rates based on long-term data is clearly lacking in our study system to support the existence of an attractive sink, even if we demonstrated a mismatch between habitat preference and some important fitness components.

A possible explanation for the observed decision-making pattern in the Red-backed shrike is that early-successional vegetation in Spruce plantations shares some features with the open areas created by natural disturbance regimes in forest [30], [32], [66], [67] and shrikes (and other bird species) probably used these features in the past to guide their settlement decisions (i.e. genetically inherited cues, see [9], [68]). Although they can imitate ancestrally used habitats, current forest management practices may also impact important resources and conditions in such a way that habitat quality for the species is much lower in artificial early-successional regeneration areas than in the naturally disturbed areas. In particular, reforestation (i.e. tree planting) or post-disturbance logging may shorten the duration of the system, influence microclimate conditions, modify vegetation structure or eliminate some biological legacies such as woody debris or other organically derived structures [49]. Weldon and Haddad showed, for instance, that the disturbance-dependent bird species, the Indigo Bunting (*Passerina cyanea*), is attracted by habitat edges associated with increased predation pressure, which results in decreased fitness along edgy environments [69]. The authors suggested that, even though current forest management might create vegetation structures that share some similarities from the bunting's perspective with the historically used habitats, this attraction is detrimental in terms of fitness. Similarly, nest failures for the shrikes were more prevalent in the habitat associated with a higher preference. As the vegetation surrounding the farmland and woodland sites is structurally different, with a higher amount of forest edges close to the woodland sites, this may induce a contrasting level of on-nest predation pressure [70]. Alternatively but not exclusively, nests are located in different vegetation structures in farmland sites (i.e. thorny shrubs) compared to woodland sites (i.e. young trees of Spruce or Black Elder), which may be significant for differences in nest concealment and anti-predation sheltering.

Other candidate habitat selection cues in the case of the Red-backed shrike may relate to food availability. Hromada and colleagues [13] have recently shown that larders (i.e. storing of prey items) of the Great-grey shrike (*Lanius excubitor*) are used as cues by males of Red-backed shrike to evaluate habitat quality and to rapidly trigger the territory establishment after migration arrival in springtime. Future work in our study system should, therefore, also analyse potential differences in the availability, use and nutritional quality of food resources between the habitat types during the whole breeding season. This may contribute to the proximate factors explaining the differences in habitat preference and reproductive performance between farmland and woodland sites.

In summary, this study on a long-distance migrant and territorial bird in a mosaic of farmland and woodland habitats indicates that the newly colonised forest environment may be a less favourable habitat in contrast to what has been assumed previously based on presence/absence and abundance data only [26], [48]. A compound estimate of fitness based on long-term reproduction and survival data is, however, needed if we are to evaluate the significance of maladaptive individual decisions for the population dynamics in the landscape. Our study illustrates the need for a proximate understanding of the processes of habitat selection in farmland bird species that expand their breeding sites to other human-created habitats like early-successional vegetation following harvesting activities in Spruce plantations. It more generally demonstrates the significance of integrating knowledge on landscape-level behavioural processes with conservation and landscape management in dynamic environments under intense human use [71].
